# Estimated rates of influenza‐associated outpatient visits during 2001‐2010 in 6 US integrated healthcare delivery organizations

**DOI:** 10.1111/irv.12495

**Published:** 2018-02-15

**Authors:** Hong Zhou, William W. Thompson, Edward A. Belongia, Ashley Fowlkes, Roger Baxter, Steven J. Jacobsen, Michael L. Jackson, Jason M. Glanz, Allison L. Naleway, Derek C. Ford, Eric Weintraub, David K. Shay

**Affiliations:** ^1^ Centers for Disease Control & Prevention Atlanta GA USA; ^2^ Marshfield Clinic Research Foundation Marshfield WI USA; ^3^ Kaiser Permanente Vaccine Study Center Oakland CA USA; ^4^ Department of Research and Evaluation Kaiser Permanente Southern California Pasadena CA USA; ^5^ Group Health Research Institute Seattle WA USA; ^6^ Institute for Health Research Kaiser Permanente Denver CO USA; ^7^ Center for Health Research Kaiser Permanente Northwest Portland OR USA

**Keywords:** electronic health records, human, influenza, office visits, statistical models

## Abstract

**Background:**

Population‐based estimates of influenza‐associated outpatient visits including both pandemic and interpandemic seasons are uncommon. Comparisons of such estimates with laboratory‐confirmed rates of outpatient influenza are rare.

**Objective:**

To estimate influenza‐associated outpatient visits in 6 US integrated healthcare delivery organizations enrolling ~7.7 million persons.

**Methods:**

Using negative binomial regression methods, we modeled rates of influenza‐associated visits with ICD‐9‐CM‐coded pneumonia or acute respiratory outpatient visits during 2001‐10. These estimated counts were added to visits coded specifically for influenza to derive estimated rates. We compared these rates with those observed in 2 contemporaneous studies recording RT‐PCR‐confirmed influenza outpatient visits.

**Results:**

Outpatient rates estimated with pneumonia visits were 39 (95% confidence interval [CI], 30‐70) and 203 (95% CI, 180‐240) per 10 000 person‐years, respectively, for interpandemic and pandemic seasons. Corresponding rates estimated with respiratory visits were 185 (95% CI, 161‐255) and 542 (95% CI, 441‐823) per 10 000 person‐years. During the pandemic, children aged 2‐17 years had the largest increase in rates (when estimated with pneumonia visits, from 64 [95% CI, 50‐121] to 381 [95% CI, 366‐481]). Rates estimated with pneumonia visits were consistent with rates of RT‐PCR‐confirmed influenza visits during 4 of 5 seasons in 1 comparison study. In another, rates estimated with pneumonia visits during the pandemic for children and adults were consistent in timing, peak, and magnitude.

**Conclusions:**

Estimated rates of influenza‐associated outpatient visits were higher in children than adults during pre‐pandemic and pandemic seasons. Rates estimated with pneumonia visits plus influenza‐coded visits were similar to rates from studies using RT‐PCR‐confirmed influenza.

## 1 INTRODUCTION

Influenza infections are responsible for substantial morbidity during most seasons.^1^, ^2^, ^3^, ^4^, ^5^, ^6^, ^7^ Influenza‐associated illnesses are difficult to count because symptoms are non‐specific, diagnostic codes associated with influenza‐related symptoms are broad, and sensitive and specific laboratory testing for influenza is not routine. Many studies have estimated rates of serious complications of influenza infections—including hospitalizations and deaths—with statistical models.^1^, ^2^, ^3^, ^4^, ^5^, ^6^, ^7^, ^8^, ^9^, ^10^, ^11^ Modeling these outcomes is routine because severe influenza sequelae are uncommon, and confirmation of infection in such patients may be difficult, even with modern diagnostics. By contrast, influenza‐associated outpatient visits are plentiful, so rates of laboratory‐confirmed visits should be easier to document. However, relatively few studies have made population‐based estimates of influenza‐confirmed outpatient visit rates.^12^, ^13^, ^14^, ^15^, ^16^, ^17^ Most have focused on children, typically in a single site.^12^, ^14^, ^15^, ^16^, ^17^ Often, few influenza‐confirmed cases are reported (range, 90‐372),^12^, ^13^, ^15^, ^17^ resulting in wide confidence intervals (CIs) around rate estimates. While a key characteristic of influenza is season‐to‐season variability in intensity and severity, most studies have focused on a few seasons,^12^, ^13^, ^17^, ^18^ or reported summary estimates from multiple seasons.^14^, ^15^, ^16^ Finally, studies testing for influenza per protocol are not common,^12^, ^13^, ^15^, ^17^ as prospective studies are resource‐intensive. Thus, such studies are rarely conducted in large populations including persons of all ages, in multiple sites, or during multiple influenza seasons.

The 2009 influenza A(H1N1) pandemic highlighted a lack of US population‐based rates of medically attended influenza‐associated illnesses. The Centers for Disease Control and Prevention (CDC) and National Institute of Health estimated pandemic‐associated illnesses, hospitalizations, and deaths,^19^, ^20^, ^21^, ^22^ but few estimates of the incidence of influenza‐like outpatient illnesses associated with H1N1pdm09 infection are available.^23^ Without consistently made estimates of influenza‐related outpatient visits, the complete health burden of influenza cannot be established.

We used electronic health data from 6 integrated healthcare delivery organizations (hereafter, sites) participating in the CDC‐funded Vaccine Safety Datalink (VSD) project to estimate rates of influenza‐associated outpatient visits. Our analysis included the 2009 pandemic and the 8 preceding influenza seasons. We compared several estimates of influenza‐associated outpatient rates with those derived from 2 concurrently conducted studies testing for influenza with reverse‐transcription polymerase chain reaction (RT‐PCR) assays.

## 2 METHODS

### 2.1 Study population

The VSD was established in 1990 to monitor vaccine safety in the US childhood immunization program.^24^, ^25^ It has since expanded in size and scope. Currently, there are 8 participating integrated healthcare delivery systems that enroll about 10 million persons of all ages, or ~3% of the US population. Standardized data files with demographic information, enrollment history, healthcare utilization, and mortality data are maintained at each participating site, and accessed via a distributed data model to ensure confidentially; data quality checks are performed weekly to evaluate the quality of vaccination and medical encounter data.^26^ An assessment of possible differences between the insured VSD population and the overall US population found no substantial differences by sex, race, ethnicity, or educational attainment; adults aged 55 through 64 years were slightly over‐represented in VSD data.^27^ A review of active vaccine adverse event detection systems noted the pioneering role of the VSD, how it has served as a model for systems in other countries, and its continuing innovation in data management and study design.^28^

Individuals enrolled in 6 VSD sites during 2001‐10—Kaiser Permanente Northern California, Kaiser Permanente Colorado, Kaiser Permanente Northwest (Oregon), Kaiser Permanente Southern California, Marshfield Clinic, and Group Health Cooperative—constituted the study population. Study data included demographic and medical information for each enrollee, including age, sex, enrollment dates, vaccination dates, and International Classification of Diseases, Ninth Revision, Clinical Modification (ICD‐9‐CM) diagnosis codes assigned to medical encounters, including those in outpatient settings. Outpatient settings included clinics, urgent care clinics, and emergency departments.

### 2.2 Human subjects

Institutional review boards at each of the 6 sites reviewed and approved the study protocol.

### 2.3 Viral surveillance data

Viral data from WHO Collaborating laboratories and National Respiratory and Enteric Virus Surveillance System (NREVSS) laboratories located within the 3 US regions (East North Central, Mountain, and Pacific) that contained the study sites were collected from 2001‐02 through 2009‐10. Laboratories reported weekly the number of influenza tests performed and the number positive for A(H1N1), A(H3N2), and B viruses. The first positive test for A(H1N1)pdm09 was reported during the week ending April 25, 2009,^29^ when seasonal influenza isolates were still predominant. A(H1N1)pdm09 became the predominant virus during week 17, ending May 2, 2009. We defined the pandemic as beginning week 17 of 2009, and ending week 26 of 2010. Weekly respiratory syncytial virus (RSV) data were obtained from NREVSS laboratories during the study period. There were 69‐238 hospital‐based, public health, and free‐standing laboratories located in 38‐47 states. They reported weekly the number of specimens tested for RSV by antigen detection and viral isolation methods and the number of positive results.^30^

### 2.4 ICD‐9‐CM‐coded outpatient visits

We analyzed weekly outpatient visits listing ICD‐9‐CM codes for pneumonia (480‐486), influenza (487‐488), or respiratory diseases (460‐519). If a patient had 2 or more visits listing a code of interest within 7 days, then only the first visit was used in analyses. Data were stratified into 5 age groups (<2, 2‐17, 18‐49, 50‐64, and ≥65 years) for comparison purposes.

### 2.5 Models for estimating influenza‐associated outpatient visits

We fit age‐ and site‐specific negative binomial regression models to weekly outpatient visits coded for pneumonia or respiratory diseases.^5^, ^6^, ^7^ We considered visits coded specifically for influenza to represent acute influenza infections; thus, visits listing 487‐488 were not included in regression models. Data for the proportions of specimens testing positive by week for A(H1N1), including H1N1pdm09 and seasonal H1N1, A(H3N2), and B viruses, were included in all models. A model can be summarized as:

$$\begin{array}{cc} Y_{\mathrm{age}-\;\mathrm{site}}\left(i\right) & \;=\;\alpha \exp\{\beta_{0}+\beta_{1}\left[t_{i}\right]\;+\;\beta_{2}\left[t_{i}^{2}\right]\;+\;\beta_{3}\left[\sin\left(2t_{i}\pi /52.15\right)\right]\\ & \;\;+\;\beta_{4}\left[\cos\left(2t_{i}\pi /52.15\right)\right]\;+\;\beta_{5}\left[\text{A(H1N1)}\right]\;+\;\beta_{6}\left[\text{A(H3N2)}\right]\\ & \;\;+\;\beta_{7}\left[B\right]\;+\;\beta_{8}\left[\text{RSV}\right]\} \end{array}$$

where *Y*_age,site_(*i*) was the predicted number of outpatient visits by age group and site during week *i*; α was the offset term, equal to the natural log of the population size for each age group and site; and β_5_ through β_8_ represented coefficients associated with a standardized estimate of the proportions of specimens testing positive for influenza or RSV during a given week in the region corresponding to a site.^7^

To estimate influenza‐associated pneumonia or respiratory, we started with visits predicted by a full model incorporating all viral terms and those predicted by a model in which an influenza covariate was set to 0, as previously described.^7^ Weekly site‐ and age‐specific numbers of estimated influenza‐associated outpatient visits for each site were the sum of predicted pneumonia or respiratory visits and visits coded specifically for influenza. Annual age‐specific incidence estimates were calculated as the sum of estimated outpatient visits in that age group divided by the sum of enrollments from the 6 participating sites. We estimated 95% confidence intervals (CIs) for each rate using 2.5th and 97.5th percentiles from a distribution derived from 10 000 bootstrap simulations.^31^

### 2.6 Comparisons of estimated influenza‐associated outpatient visits with RT‐PCR‐confirmed influenza rates

To check the validity and precision of rates estimated as described above, we compared them with rates derived in 2 studies that tested outpatients for influenza infection using RT‐PCR. Marshfield Clinic rate estimates were compared with rates obtained using data from annual influenza vaccine effectiveness (VE) studies conducted there.^32^, ^33^ In VE studies, patients presenting with acute respiratory symptoms were approached for enrollment; if consented, a respiratory specimen was collected and tested with CDC‐approved real‐time RT‐PCR assays. Data on RT‐PCR‐confirmed influenza visits were available among persons aged ≥50 years from 2005‐06 through 2009‐10; similar data were not available from other sites. Our pandemic rates were compared with those derived from a US influenza surveillance system, the Influenza Incidence Surveillance Project (IISP). IISP began during the 2009 pandemic. It conducted influenza surveillance in 38 outpatient practices in Florida, Iowa, Minnesota, North Dakota, Utah, and Wisconsin and New York City.^22^ Estimates of the incidence of influenza‐confirmed influenza‐like illness (ILI) outpatient visits among 272 642 outpatients were made from October 2009 through July 2012. The number of influenza‐associated ILI cases each week was estimated by multiplying the proportion of ILI visits testing positive for influenza by RT‐PCR and the number of ILI patient visits reported during each week. Incidence rates were calculated by dividing numbers of influenza‐associated ILIs by age‐specific denominators representing the outpatient practice population.^23^

## 3 RESULTS

From 2001‐02 through 2008‐09, an annual mean of 31 092 specimens (range, 20 145‐48 798) was tested for influenza in US regions that included the participating sites (Table 1). Of these, 16.5% tested positive for non‐pandemic viruses. By type and subtype, these proportions were 3.6%, 8.7%, and 4.2% for A(H1N1), A(H3N2), and B viruses, respectively. A total of 171 545 specimens were tested during the pandemic period, and 48 005 (28.0%) tested positive for A(H1N1)pdm09 virus.

**Table 1:** Annual influenza surveillance virus data for pre‐pandemic (2001‐02 through 2008‐09) and pandemic seasons in 3 US regions^a^

Season	Years	No. of specimens Tested	Positive tests for non‐pandemic viruses								Positive tests for A(H1N1)pdm09 virus	
			A (H1N1)		A (H3N2)		B		Total			
			N	%	N	%	N	%	N	%	N	%
Non‐pandemic	2001‐02	21 453	58	0.3	2636	12.3	744	3.5	3438	16.0	—	—
	2002‐03	20 145	1519	7.5	602	3.0	819	4.1	2940	14.6	—	—
	2003‐04	30 882	0	0.0	5273	17.1	58	0.2	5331	17.3	—	—
	2004‐05	32 172	4	0.0	3231	10.0	2031	6.3	5266	16.4	—	—
	2005‐06	32 576	122	0.4	3582	11.0	1151	3.5	4855	14.9	—	—
	2006‐07	48 798	2101	4.3	2022	4.1	986	2.0	5109	10.5	—	—
	2007‐08	35 294	1963	5.6	3113	8.8	2394	6.8	7470	21.2	—	—
	2008‐09	27 415	2997	10.9	969	3.5	1918	7.0	5884	21.5	—	—
	Annual mean	31 092	1095	3.6	2679	8.7	1263	4.2	5037	16.5	—	—
Pandemic	2009‐10	171 545	764	0.4	2521	1.5	663	0.4	3948	2.3	48 005	28.0

a The East North Central, Mountain, and Pacific US regions in which the 6 participating health systems provided care.

The 6 sites enrolled ~7.7 million persons annually during the study period. An annual mean rate of 28 (95% CI, 18‐53) outpatient visits per 10 000 person‐years was coded specifically for influenza (Table 2). For pneumonia/influenza and respiratory outpatient visits, the annual mean rates were 231 (95% CI, 206‐374) and 4846 (95% CI, 4597‐6205) visits per 10 000 person‐years, respectively. The highest annual mean rate of influenza visits occurred among persons aged 2‐17 years (46, 95% CI, 32‐98); the lowest rate occurred among persons aged ≥65 years (10, 95% CI, 6‐22). Rates for pneumonia/influenza and respiratory outpatient visits were highest among children aged <2 years (676, 95% CI, 588‐968, and 15 385, 95% CI, 13 696‐17 673, respectively), and were lowest among persons aged 18‐49 years (110, 95% CI, 93‐191, and 3819, 95% CI, 3597‐4963, respectively).

**Table 2:** Annual rates of outpatient visits per 10 000 person‐years for 3 categories of respiratory illnesses, by age group, among 6 US healthcare delivery systems

Season	Year	Age group (y)																	
		<2			2‐17			18‐49			50‐64			≥65			All ages		
		Rate	95%	CI	Rate	95%	CI	Rate	95%	CI	Rate	95%	CI	Rate	95%	CI	Rate	95%	CI
Influenza^a^																			
Non‐pandemic	2001‐02	28	13	117	28	15	80	19	8	42	13	6	24	6	4	16	18	9	42
	2002‐03	24	10	78	34	13	90	17	6	29	11	4	18	5	2	9	18	7	39
	2003‐04	90	49	334	62	33	147	34	17	69	25	11	54	18	9	51	37	19	87
	2004‐05	29	14	137	29	16	107	22	12	79	18	9	60	9	4	33	21	11	72
	2005‐06	35	26	89	45	26	97	24	15	38	18	11	26	9	5	15	25	15	44
	2006‐07	33	24	60	45	41	74	22	20	35	15	12	22	6	4	11	23	21	35
	2007‐08	59	43	125	70	63	105	63	47	89	45	32	58	16	12	31	53	42	71
	2008‐09	26	21	49	51	46	85	29	21	39	18	13	24	7	5	9	28	23	36
	Annual mean	41	25	124	46	32	98	29	18	53	20	12	36	10	6	22	28	18	53
Pandemic	2009‐10	276	237	501	325	310	431	166	140	198	105	83	126	40	31	49	173	154	207
Pneumonia and influenza																			
Non‐pandemic	2001‐02	605	512	1018	266	236	446	103	78	202	197	165	331	427	362	800	216	187	390
	2002‐03	606	509	938	254	213	381	92	79	164	189	165	323	423	351	812	208	181	372
	2003‐04	696	588	1154	287	261	392	113	89	205	212	173	362	462	378	853	237	208	411
	2004‐05	650	553	1042	298	257	452	102	84	232	203	169	397	441	365	826	230	195	429
	2005‐06	771	674	959	360	324	421	115	106	180	222	198	326	463	389	696	258	242	364
	2006‐07	705	607	886	286	245	364	103	95	161	200	179	289	418	357	672	225	207	339
	2007‐08	787	703	1051	324	288	455	154	132	237	252	217	334	452	374	696	272	247	400
	2008‐09	591	558	697	268	250	335	96	79	144	175	142	254	349	289	549	200	179	291
	Annual mean	676	588	968	293	259	406	110	93	191	206	176	327	430	358	738	231	206	374
Pandemic	2009‐10	1214	1136	1439	765	727	850	291	248	387	368	299	488	664	506	1009	481	431	617
Respiratory diseases																			
Non‐pandemic	2001‐02	17 646	15 032	20 971	6354	5860	8221	3760	3524	5011	4270	4056	5259	4323	3978	6441	4820	4607	6097
	2002‐03	17 269	14 516	20 494	6204	5490	7469	3939	3782	4894	4447	4202	5696	4593	3973	7781	4913	4620	6514
	2003‐04	15 811	13 558	18 607	5469	4946	6459	3759	3537	4982	4361	4016	5906	4802	4109	8115	4659	4423	6158
	2004‐05	15 207	13 197	17 733	5545	4960	7238	3745	3479	5400	4441	4145	6132	4979	4320	8637	4696	4344	6556
	2005‐06	15 567	14 008	17 688	5919	5407	7423	3946	3732	5123	4669	4468	5820	5246	4664	7806	4953	4671	6335
	2006‐07	15 079	13 905	16 473	5314	5036	6835	3852	3709	4908	4875	4670	5685	5718	5019	7657	4899	4647	6238
	2007‐08	14 844	14 204	16 166	5497	5131	7247	4197	3892	5035	5562	5262	5802	6608	5704	8267	5361	5165	6328
	2008‐09	11 657	11 152	13 254	4740	4415	5972	3355	3124	4350	4612	4319	5165	5733	4855	6871	4466	4298	5412
	Annual mean	15 385	13 696	17 673	5630	5155	7108	3819	3597	4963	4655	4392	5683	5250	4578	7697	4846	4597	6205
Pandemic	2009‐10	16 469	15 815	17 962	7446	6996	9009	5313	5016	6895	7275	6560	8678	11 541	9669	14 098	7283	6880	8806

Rate is calculated as the sum of estimated outpatient visits divided by the sum of enrollments from 6 participating sites.

CI, denotes confidence interval generated by bootstrap simulation.

Specific ICD‐9‐CM codes for influenza, pneumonia, and respiratory illness categories provided in the Methods.

During the 2009 pandemic, rates for influenza, pneumonia/influenza, and respiratory visits were 173 (95% CI, 154‐207), 481 (95% CI, 431‐617), and 7283 (95% CI, 6880‐8806) per 10 000 person‐years, respectively (Table 2). The highest rates for outpatient visits coded for influenza occurred in children aged 2‐17 years, followed by children aged <2 years; the lowest rates occurred among persons aged ≥65 years. Rates for pneumonia/influenza and respiratory outpatient visits were highest among children aged <2 years, followed by persons aged 2‐17 years or 65 years and older, while the lowest rate was among adults aged 18‐49 years.

### 3.1 Estimates of influenza‐associated outpatient visits

Among persons of all ages, the annual mean rate of influenza‐associated visits estimated with models using pneumonia‐coded visits was 39 (95% CI, 30‐70) per 10 000 person‐years during 2001‐02 through 2008‐09. It was 203 (95% CI, 180‐240) per 10 000 person‐years during the 2009 pandemic (Table 3). During non‐pandemic and pandemic seasons, rates were higher among younger persons. In pre‐pandemic seasons, the highest rate was 67 (95% CI, 49‐164) per 10 000 person‐years in children aged <2 years. The lowest rate was 25 (95% CI, 20‐48) for persons aged ≥65 years. The highest rate during the pandemic was 381 (95% CI, 366‐481) in children aged 2‐17 years; the lowest rate was 63 (95% CI, 56‐86) per 10 000 person‐years for individuals aged ≥65 years. We found variability by site as well as by age group in estimated rates, but as no consistent patterns of geographic variability were noted (Table S1), we focused on the clear variability in rates by age group.

**Table 3:** Annual rates of influenza‐associated outpatient visits per 10 000 person‐years estimated with negative binomial regression models, by age group, among 6 US healthcare systems

Season	Year	Age groups (y)																	
		<2			2‐17			18‐49			50‐64			≥65			All		
		Rate	95%	CI	Rate	95%	CI	Rate	95%	CI	Rate	95%	CI	Rate	95%	CI	Rate	95%	CI
Pneumonia and Influenza^a^																			
Non‐Pandemic	2001‐02	63	46	176	46	36	101	24	13	52	23	15	42	27	22	54	30	21	62
	2002‐03	41	27	117	54	36	123	23	13	39	20	15	33	17	14	31	29	19	56
	2003‐04	123	83	374	78	50	157	38	20	76	33	18	66	37	25	83	48	29	101
	2004‐05	56	37	182	49	35	137	29	19	92	30	21	80	28	20	68	34	24	94
	2005‐06	71	62	125	64	49	116	29	20	46	28	21	40	28	23	42	37	27	58
	2006‐07	56	45	82	60	55	90	26	23	41	22	18	29	16	14	23	31	29	45
	2007‐08	90	67	197	96	85	140	70	56	99	57	47	72	33	28	61	67	59	94
	2008‐09	33	27	62	63	55	101	33	25	43	24	20	29	13	11	22	34	30	46
	Annual mean	67	49	164	64	50	121	34	24	61	30	22	49	25	20	48	39	30	70
Pandemic	2009‐10	302	259	543	381	366	481	186	155	222	131	103	159	63	56	86	203	180	240
Respiratory diseases																			
Non‐pandemic	2001‐02	352	285	517	395	341	451	141	115	212	136	122	187	79	65	99	191	170	247
	2002‐03	197	164	411	437	408	591	156	140	245	127	97	218	56	38	129	197	180	298
	2003‐04	377	338	543	231	161	296	99	49	154	100	65	136	76	55	97	130	84	175
	2004‐05	297	210	715	564	509	727	192	161	348	186	134	328	93	58	141	256	219	384
	2005‐06	302	271	387	336	304	381	134	112	181	140	128	159	88	70	94	173	155	202
	2006‐07	168	160	201	219	198	279	96	82	140	90	78	112	54	48	67	115	103	147
	2007‐08	261	215	511	544	516	732	235	214	370	221	176	317	117	75	182	277	258	388
	2008‐09	97	75	242	278	266	347	118	106	180	109	69	171	59	27	112	139	123	197
	Annual mean	256	215	441	375	338	476	146	122	229	139	109	204	78	54	115	185	161	255
Pandemic	2009‐10	460	367	763	954	817	1108	505	414	751	410	297	758	219	116	697	542	441	823

Rate is calculated as the sum of estimated outpatient visits divided by the sum of enrollments from 6 participating sites.

CI, denotes confidence interval generated by bootstrap simulation.

Specific ICD‐9‐CM codes for influenza, pneumonia, and respiratory illness categories provided in the Methods.

The annual mean rate of influenza‐associated visits estimated with models using respiratory‐coded visits was 185 (95% CI, 161‐255) per 10 000 person‐years during pre‐pandemic seasons and 542 (95% CI, 441‐823) per 10 000 person‐years during the pandemic (Table 3). During non‐pandemic seasons, the highest rate was 375 (95% CI, 338‐476) per 10 000 person‐years in children aged 2‐17 years, while the lowest rate was 78 (95% CI, 54‐115) in persons aged ≥65 years. Similarly, during the pandemic, the highest rate was in children aged 2‐17 years and the lowest rate was in persons aged ≥65 years.

**Figure 1:**
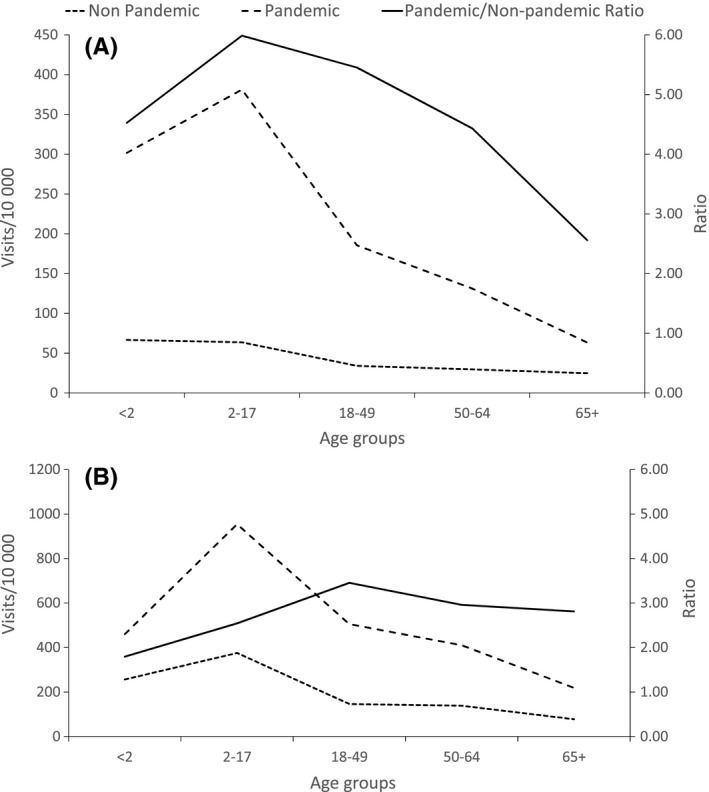
Estimated rates of outpatient visits in 6 US healthcare delivery organizations (by age group) per 10 000 person‐years (left *y*‐axis) for influenza‐associated pneumonia and influenza (Panel A) and respiratory diseases (Panel B); and pandemic‐to‐pre‐pandemic rate ratios (right *y*‐axis): solid line, rate ratio; dashed line, pandemic rate; and dotted line, pre‐pandemic rate

Age‐specific rates during the pandemic and pre‐pandemic seasons and incidence rate ratios are provided in Figure 1 for estimates made with pneumonia‐coded (Panel A) or respiratory‐coded (Panel B) visits. Substantial increases occurred during the pandemic, most prominently among younger persons. For estimates made with pneumonia visits, the pandemic‐to‐pre‐pandemic ratios were 5.5, 6.0, and 4.5 among persons aged 18‐49 years, 2‐17 years, and <2 years, respectively. The lowest ratio was 2.6, among persons aged ≥65 years. For estimates made with respiratory visits, a substantial increase in outpatient rates during the pandemic was also noted, although it was smaller. The greatest pandemic/pre‐pandemic ratio was 3.5 among persons aged 18‐49 years (Panel B).

### 3.2 Rate comparisons

**Figure 2:**
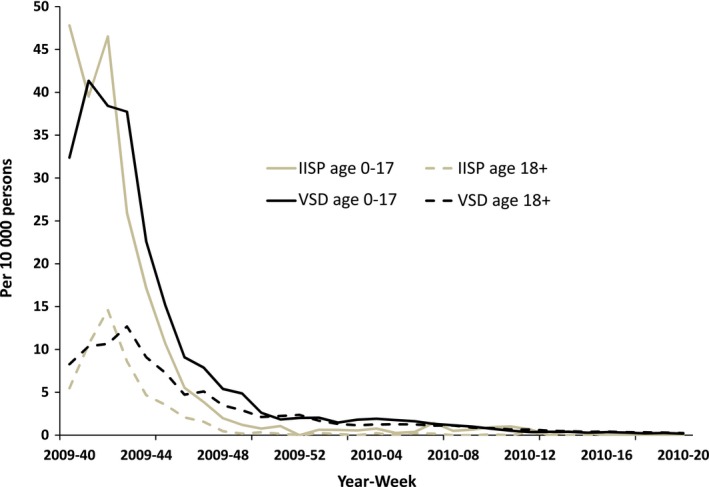
Estimated weekly rates of outpatient visits per 10 000 persons for influenza‐associated pneumonia and influenza in 6 US healthcare delivery organizations participating in the Vaccine Safety Datalink (VSD) and for influenza‐associated influenza‐like illness outpatient visits from in the Influenza Incidence Surveillance Project (IISP), from October (week 40) 2009 through April (week 20) 2010

Our estimates of influenza‐associated outpatient visits among persons aged ≥50 years at Marshfield Clinic were similar to rates of RT‐PCR‐confirmed influenza visits calculated with VE study data (Table 4). Rates of RT‐PCR‐confirmed influenza visits each season fell within the 95% CIs of our model‐based estimates in 4 of 5 seasons. During the mild 2005‐06 season, our estimated rate made with pneumonia visits was significantly greater than the rate from the VE study. During the pandemic, rates estimated with pneumonia were similar to rates of RT‐PCR‐confirmed Influenza among subjects enrolled in the IISP. Figure 2 plots our estimated weekly rates and weekly rates of influenza‐associated ILI from IISP. The timing, peak, and magnitude of these rates were consistent among both children aged 0‐17 years and adults aged 18 and older.

**Table 4:** Annual influenza‐associated outpatient visits per 10 000 persons aged ≥50 y at Marshfield Clinic, estimated (i) using negative binomial models with pneumonia and influenza data; and (ii) using RT‐PCR‐confirmed influenza outpatient visits in annual influenza vaccine effectiveness studies

Season	Negative binomial model‐based estimates			RT‐PCR‐based estimates
	95% confidence intervals			
	Estimate	Lower^a^	Upper	
2005‐06	34.7	27.6	119.0	20.3
2006‐07	24.3	23.0	105.0	28.5
2007‐08	125.7	108.3	241.9	150.5
2008‐09	21.4	21.4	94.1	25.8
2009 (weeks 40‐44)	40.3	40.3	52.7	39.9

a The lower confidence limit is defined by a rate established by visits listing the ICD‐9‐CM code for influenza.

## 4 DISCUSSION

We estimated influenza‐associated outpatient visit rates in 6 US healthcare organizations enrolling ~7.7 million persons and found that rates were greater among children and young persons during pre‐pandemic and pandemic influenza seasons. The 2009‐10 pandemic was associated with significant increases in rates of influenza‐associated outpatient visits in each of 5 age groups when compared with rates from preceding seasons. These increases were most pronounced in children, as expected.

This pattern of results is consistent with findings from studies of hospitalizations and deaths. One study estimating laboratory‐confirmed US hospitalizations during the 2009‐10 pandemic^22^ found that the overall ratio of pandemic to seasonal hospitalizations was 1.7, whereas among persons aged 18‐64 years, the ratio was 4.0, and among children aged 0‐17 years, it was 7.4. Among persons aged ≥65 years, rates of hospitalizations and deaths during the pandemic^22^, ^34^ were low when compared with seasonal influenza‐associated outcomes.^5^, ^6^, ^7^ Viboud et al^20^ suggested that the mean age at death of 37 years during the 2009‐10 pandemic meant that the estimated years of life lost during 2009 alone exceeded those lost during the 1968 pandemic, when the mean age at death was 62 years. Cross‐protective immunity in older persons from prior infections with H1N1 viruses more closely related antigenically to the pandemic strain than H1N1 viruses circulating since 1977 contributed to these findings.^35^

We estimated influenza‐associated outpatient visits by adding influenza‐coded visits to model‐based estimates of influenza‐associated visits in 2 categories: a more restrictive category of outpatient visits coded for pneumonia, and a broader category of visits coded for acute respiratory illnesses. Both categories have been used in studies modeling the burden of influenza‐related illnesses.^5^, ^6^, ^7^, ^16^, ^17^ It is likely that use of the pneumonia category underestimates the outpatient burden of influenza, because influenza illnesses may be coded for with a variety of ICD‐9‐CM codes, including codes associated with bacterial infections, like bronchitis or sinusitis. It has been proposed that estimates of influenza‐associated pneumonia deaths represent a lower bound of the range of deaths related to influenza infections,^6^, ^20^ because influenza‐related deaths may represent exacerbations of chronic pulmonary diseases as well. This rationale may also apply to less severe influenza‐associated medical encounters, and therefore, we also made estimates of all respiratory visits that may be related to influenza infections.

Most modeling‐based estimates of serious influenza‐related outcomes (particularly of deaths) cannot be verified because a “gold standard” for laboratory‐confirmed late sequelae is lacking. By contrast, we compared our estimated outpatient rates with those made in 2 studies that prospectively enrolled and tested outpatients for influenza infection with RT‐PCR (ie, gold standard) methods. Our estimates of outpatient rates made using pneumonia‐coded visits in adults aged ≥50 years at Marshfield Clinic were compared with those derived from a series of annual VE studies conducted there. Our rates were statistically consistent with rates of RT‐PCR‐confirmed influenza visits during 4 of 5 seasons. During the mild 2005‐06 influenza season, when few patients enrolled in Marshfield's VE study,^33^ our model‐based estimate of 35 visits per 10 000 person‐years was significantly greater than the RT‐PCR‐confirmed estimate of 20 visits per 10 000 person‐years. There are several possible reasons for this finding. First, because of the relative paucity of influenza activity during 2005‐06, we may have attributed illnesses to influenza that were associated with other acute respiratory pathogens. Most patients tested for influenza with RT‐PCR at Marshfield Clinic were recruited by staff based on chief complaints of fever or respiratory symptoms, and not on the basis of ICD‐9‐CM diagnosis codes recorded after medical care. It is possible also that during milder influenza seasons, ICD codes may be less useful for identifying influenza cases. Based on these comparisons, we believe that future modeling studies should attempt to include more mild influenza seasons, as data from a broad range of seasons should permit better calibration of statistical models, and prevent possible overestimation of influenza‐mediated events.

The second source of PCR‐confirmed data was limited to the pandemic season. Estimated rates based on pneumonia‐coded visits in the 6 participating sites were broadly consistent with rates of RT‐PCR‐confirmed influenza in the IISP study, which tested outpatients presenting with ILI.^23^ As the ILI syndrome likely does not detect all illnesses that may be associated with influenza infections, this PCR‐based estimate is conservative, suggesting that our pneumonia‐based estimates are also conservative. Data from studies using sensitive diagnostics among a more inclusive set of signs and symptoms of influenza would be helpful.

More model‐based estimates of influenza‐associated outpatient visits should be compared with data from studies using RT‐PCR or other highly sensitive diagnostics. The generalizability of this study's findings (and its underlying models) is limited by the paucity of comparison data. For example, the pandemic season was unusual—increased healthcare utilization consistent with intense media coverage was noted in 1 study site^36^—and comparisons made then may not be applicable during interpandemic periods. In Marshfield, comparison data were available only for older adults. Overall, our results suggest that models developed for more severe influenza outcomes yield influenza outpatient rate estimates that appear consistent with rates calculated with data collected in protocol‐based studies using state‐of‐the‐art diagnostics.

Our estimated rates of influenza‐associated outpatient are somewhat lower than rates reported in some other studies. Poehling et al^17^ reported that among children aged <2 years in 3 sites, the incidence of outpatient visits attributable to influenza among children was 280‐520 per 10 000 during 2002‐03 and 590‐1250 per 10 000 during 2003‐04. For the same age group, we estimated influenza‐associated respiratory visit rates were 197 per 10 000 and 377 per 10 000 in these 2 seasons, respectively. Besides the usual caveats regarding differences in study design affecting incidence estimates, it is possible that geographic differences in influenza activity also affected these comparisons. Poehling's study had 3 sites and ours 6; neither had the population size or geographic variation for its estimates to be interpreted as national in scope. We did find variation among our sites in estimated rates (Table S1); however, no clear age‐specific patterns of differences by geography were apparent. Although estimated rates may be difficult to compare because of differences in study design, populations studied, seasons included, and geography, rate ratios between age groups should be similar. Here our findings are consistent with those from other studies^12^, ^14^, ^15^, ^16^, ^17^, ^23^: Young children bear the brunt of the outpatient influenza burden. Finally, although our study included >7 million persons, a larger population than other US studies, all data were from integrated health systems. Thus, at least with respect to age, our population is unlikely to fully represent the US population.^27^ The generalizability of our findings to other US populations, like the uninsured and those covered by the Department of Veterans Affairs, may be more limited.

In addition to the issues noted above, we acknowledge other limitations. While we did adjust for RSV activity by including a weekly term for this virus in all models, we could not consider the effects of other viral or bacterial respiratory pathogens on influenza estimates. Because RSV circulation often overlaps with influenza circulation and it is the leading cause of infectious respiratory disease among in young children,^6^, ^37^ we emphasized adjusting for regional RSV activity. Because the onset, duration, and intensity of influenza virus circulation vary geographically,^38^ aggregating virus surveillance data by the US region of each site may not adequately capture the variability in timing of influenza virus circulation.

Few studies have estimated annual rates of laboratory‐confirmed influenza outpatient visits. None cover the full age spectrum, include many influenza seasons, and represent national‐level populations. Because prospective studies that consent and enroll subjects are resource‐intensive, we suggest that modeling methods similar to those used here deserve further exploration and validation for use in estimating outpatient influenza rates. These methods are less expensive and may provide reasonably sensitive, specific, and timely estimates of the influenza‐associated outpatient disease burden. Regular assessments of the burden of annual epidemics and occasional pandemics are crucial for quantifying the potential benefits of influenza prevention and treatment modalities over time. Such assessments need to include influenza‐associated outpatient medical visits in addition to hospitalization and deaths.

## CONFLICT OF INTEREST

R.B. received research grants from Novartis, Sanofi Pasteur, GSK, MedImmune LLC, and Protein Sciences. A.L.N. received research support from GSK. E.A.B. has received research support from MedImmune LLC. The following authors report that they do not have a commercial or other association that may pose a potential conflict of interest: H.Z., W.W.T., A.F., M.L.J., S.S.J., J.M.G., E.W., and D.K.S.

## DISCLAIMER

The findings and conclusions in this report are those of the authors and do not necessarily represent the views of the Centers for Disease Control and Prevention.
