# Supplementary material for: Estimated rates of influenza‐associated outpatient visits during 2001‐2010 in 6 US integrated healthcare delivery organizations
Source: Influenza Other Respir Viruses. 2018 Feb 15;12(1):122–31. doi: 10.1111/irv.12495 (PMC5818343; doi:10.1111/irv.12495)
Supplement: Supplementary file 1 [file IRV-12-122-s001.docx]

| **Supplemental Table.** Estimated influenza-associated pneumonia and influenza outpatient rates per 10,000 person-years, by site, season, and age group.  Age Group | | | | | | | | | | | |
| --- | --- | --- | --- | --- | --- | --- | --- | --- | --- | --- | --- |
| Site | Season | <2 yrs | | 2-17 yrs | | 18-49 yrs | | 50-64 yrs | >65 yrs | | All ages |
| Northern | 2001\2002 | 41 | 32 | | 9 | | 13 | | 22 | 217 | |
| California | 2002\2003 | 17 | 22 | | 9 | | 12 | | 14 | 13 | |
| Kaiser | 2003\2004 | 73 | 40 | | 15 | | 15 | | 21 | 22 | |
|  | 2004\2005 | 36 | 54 | | 15 | | 20 | | 29 | 27 | |
|  | 2005\2006 | 66 | 48 | | 18 | | 19 | | 23 | 6 | |
|  | 2006\2007 | 58 | 54 | | 21 | | 17 | | 12 | 26 | |
|  | 2007\2008 | 82 | 93 | | 53 | | 44 | | 29 | 56 | |
|  | 2008\2009 | 30 | 56 | | 23 | | 18 | | 11 | 27 | |
| Annual mean | | 50 | 50 | | 20 | | 20 | | 20 | 27 | |
|  | 2009\2010 | 269 | 322 | | 144 | | 93 | | 40 | 159 | |
|  |  |  |  | |  | |  | |  |  | |
| Kaiser | 2001\2002 | 117 | 61 | | 34 | | 32 | | 38 | 41 | |
| Colorado | 2002\2003 | 121 | 152 | | 47 | | 36 | | 26 | 63 | |
|  | 2003\2004 | 551 | 210 | | 101 | | 102 | | 132 | 137 | |
|  | 2004\2005 | 145 | 109 | | 73 | | 64 | | 57 | 76 | |
|  | 2005\2006 | 144 | 167 | | 46 | | 41 | | 46 | 70 | |
|  | 2006\2007 | 71 | 71 | | 30 | | 22 | | 23 | 36 | |
|  | 2007\2008 | 124 | 126 | | 75 | | 61 | | 55 | 79 | |
|  | 2008\2009 | 68 | 98 | | 34 | | 22 | | 19 | 41 | |
| Annual mean | | 168 | 124 | | 55 | | 48 | | 49 | 68 | |
|  | 2009\2010 | 642 | 527 | | 217 | | 156 | | 86 | 252 | |
|  |  |  |  | |  | |  | |  |  | |
| Marshfield | 2001\2002 | 431 | 198 | | 107 | | 70 | | 64 | 127 | |
| Clinic | 2002\2003 | 202 | 164 | | 55 | | 32 | | 21 | 78 | |
|  | 2003\2004 | 610 | 214 | | 100 | | 61 | | 63 | 132 | |
|  | 2004\2005 | 398 | 252 | | 149 | | 129 | | 95 | 168 | |
|  | 2005\2006 | 166 | 111 | | 52 | | 32 | | 38 | 63 | |
|  | 2006\2007 | 163 | 168 | | 57 | | 31 | | 22 | 76 | |
|  | 2007\2008 | 565 | 272 | | 256 | | 141 | | 112 | 222 | |
|  | 2008\2009 | 57 | 134 | | 61 | | 30 | | 15 | 64 | |
| Annual mean | | 324 | 189 | | 105 | | 66 | | 54 | 116 | |
|  | 2009\2010 | 591 | 581 | | 262 | | 126 | | 38 | 294 | |
|  |  |  |  | |  | |  | |  |  | |
| Kaiser | 2001\2002 | 71 | 93 | | 38 | | 34 | | 46 | 51 | |
| Northwest | 2002\2003 | 55 | 90 | | 23 | | 22 | | 23 | 37 | |
|  | 2003\2004 | 132 | 95 | | 34 | | 25 | | 31 | 46 | |
|  | 2004\2005 | 70 | 127 | | 51 | | 53 | | 53 | 67 | |
|  | 2005\2006 | 63 | 70 | | 27 | | 28 | | 33 | 37 | |
|  | 2006\2007 | 60 | 60 | | 24 | | 19 | | 16 | 29 | |
|  | 2007\2008 | 50 | 90 | | 40 | | 37 | | 36 | 49 | |
|  | 2008\2009 | 27 | 91 | | 26 | | 21 | | 16 | 36 | |
| Annual mean | | 66 | 89 | | 33 | | 30 | | 32 | 44 | |
|  | 2009\2010 | 306 | 344 | | 149 | | 92 | | 40 | 164 | |
|  |  |  |  | |  | |  | |  |  | |
| Southern | 2001\2002 | 45 | 41 | | 30 | | 27 | | 23 | 31 | |
| California | 2002\2003 | 35 | 52 | | 29 | | 22 | | 13 | 31 | |
| Kaiser | 2003\2004 | 95 | 90 | | 49 | | 40 | | 35 | 56 | |
|  | 2004\2005 | 36 | 36 | | 22 | | 19 | | 17 | 24 | |
|  | 2005\2006 | 66 | 68 | | 34 | | 33 | | 29 | 41 | |
|  | 2006\2007 | 39 | 49 | | 26 | | 23 | | 15 | 29 | |
|  | 2007\2008 | 57 | 79 | | 79 | | 64 | | 26 | 68 | |
|  | 2008\2009 | 24 | 48 | | 39 | | 28 | | 10 | 34 | |
| Annual mean | | 50 | 58 | | 39 | | 32 | | 21 | 39 | |
|  | 2009\2010 | 234 | 340 | | 206 | | 153 | | 62 | 208 | |
|  |  |  |  | |  | |  | |  |  | |
| Group | 2001\2002 | 115 | 79 | | 50 | | 45 | | 69 | 59 | |
| Health | 2002\2003 | 88 | 65 | | 38 | | 38 | | 44 | 46 | |
| Cooperative | 2003\2004 | 182 | 92 | | 56 | | 52 | | 57 | 65 | |
|  | 2004\2005 | 144 | 107 | | 112 | | 105 | | 107 | 109 | |
|  | 2005\2006 | 112 | 79 | | 55 | | 49 | | 53 | 59 | |
|  | 2006\2007 | 65 | 73 | | 50 | | 40 | | 27 | 47 | |
|  | 2007\2008 | 115 | 87 | | 85 | | 77 | | 62 | 79 | |
|  | 2008\2009 | 78 | 86 | | 51 | | 31 | | 27 | 47 | |
| Annual mean | | 112 | 83 | | 62 | | 55 | | 56 | 64 | |
|  | 2009\2010 | 502 | 352 | | 235 | | 152 | | 70 | 212 | |
